# Comparison of risk of complication between neuraxial anaesthesia and general anaesthesia for hip fracture surgery: a systematic review and meta-analysis

**DOI:** 10.1097/JS9.0000000000000291

**Published:** 2023-03-24

**Authors:** Xi Chen, Hairui Li, Songlin Li, Yiou Wang, Ruichen Ma, Wenwei Qian, Gang Chen, Jian Li

**Affiliations:** Departments of a Orthopedics; b Plastic Surgery, West China Hospital, West China School of Medicine, Sichuan University, Chengdu; c Department of Orthopedic Surgery, Peking Union Medical College Hospital, Peking Union Medical College, Chinese Academy of Medical Science; d School of Medicine, Tsinghua University, Beijing, China

**Keywords:** hip fracture, neuraxial anaesthesia, general anaesthesia

## Abstract

Controversy remains over the choice of anaesthetic technique for patients undergoing surgery for hip fracture.

**Aim:**

The aim was to compare the risk of complication of neuraxial anaesthesia with that of general anaesthesia in patients undergoing hip fracture surgery.

**Methods:**

This systematic review was performed according to Preferred Reporting Items for Systematic Reviews and Meta-Analysis guidelines and was registered at PROSPERO (CRD42022337384). The study included eligible randomised controlled trials published before February 2022. Data synthesis was performed to compare the differences between general and neuraxial anaesthesia. Meta-regression analysis was performed to investigate the influence of the publication year. A subgroup analysis was performed based on patient age and the anaesthetic technique used. A grading of recommendations, assessment, development and evaluations assessment was performed to assess the quality of each outcome.

**Results:**

Twenty randomised controlled trials and 4802 patients were included. Data synthesis revealed significant higher risk of acute kidney injury in the general anaesthesia group (*P*=0.01). There were no significant differences between the two techniques in postoperative short-term mortality (*P*=0.34), delirium (*P*=0.40), postoperative nausea and vomiting (*P*=0.40), cardiac infarction (*P*=0.31), acute heart failure (*P*=0.34), pulmonary embolism (*P*=0.24) and pneumonia (*P*=0.15). Subgroup analysis based on patient age and use of sedative medication did not reveal any significant differences. Meta-regression analysis of the publication year versus each adverse event revealed no statistically significant differences.

**Conclusion:**

A significantly higher risk of postoperative acute kidney injury was found in patients receiving general anaesthesia. This study revealed no significant differences in terms of postoperative mortality and other complications between general and neuraxial anaesthesia. The results were consistent across the age groups.

## Introduction

Highlights

- Twenty randomised controlled trials were included in the data synthesis.
- General anaesthesia is associated with a higher risk of acute kidney injury.
- No differences were observed in postoperative mortality and other complications.

Hip fracture is a serious injury that occurs primarily in the elderly^1^. The annual incidence of hip fracture is estimated to be over 1.6 million worldwide, with 70% of these patients exceeding the age of 80 years^2^. With 1.5–6% of patients dying from such fractures within 30 days of injury and 20% suffering from major complications, hip fractures place a huge burden on health services worldwide^3^.

Surgical treatment is required in most patients who sustain hip fractures^4^,^5^. The choice of anaesthesia technique is crucial because it significantly influences the recovery after surgery and the risk of complications, especially in older patients^6^. However, the optimal anaesthetic technique for elderly patients with hip fracture has remained controversial for decades^7^. Studies continuously focus on whether general anaesthesia or neuraxial anaesthesia is more appropriate in terms of reducing the risk of postoperative complications^8^. Potential benefits of neuraxial anaesthesia in reducing the risk of complications including delirium have been reported in previous studies^9^–^13^. However, recent randomised controlled trials found the risk of complications to be comparable between the two anaesthetic techniques^9^,^14^.

A wide variety of complications should be investigated following hip fracture surgery, such as mortality, delirium, pneumonia, pulmonary embolism, heart failure, cardiac infarction and acute kidney injury (AKI)^15^. However, most studies reported only some of the complications^16^–^20^. Additionally, the incidence of some major complications is low following hip fracture surgery and clinical trials may be underpowered to detect statistically significant differences^2^. Therefore, it is crucial to gather existing evidence from published literatures and conduct data synthesis based on a large sample size.

Meta-analyses have been published to compare the complication rate of regional and general anaesthesia in patients receiving hip fracture surgery; however, the results were inconclusive regarding the risk of postoperative complications^2^,^8^,^21^–^24^. One systematic review reported that the sample size was insufficient to draw robust conclusions from pooled analysis^7^. However, more randomised controlled trials have been published recently, including one from the REGAIN trial with 1600 participants and another one from the RAGA trial with 950 participants^9^,^14^,^17^,^18^,^20^. The inclusion of these new studies might alter the results of data synthesis in systematic reviews.

Therefore, this study aims to compare the risk of complication of neuraxial anaesthesia with that of general anaesthesia in patients undergoing hip fracture surgery.

## Methods

This systematic review and meta-analysis were conducted in accordance with the PRISMA (Preferred Reporting Items for Systematic Reviews and Meta-Analyses) and AMSTAR (assessing the methodological quality of systematic reviews) guidelines^25^,^26^. The PRISMA checklist and flowchart are available at Supplementary File 1, Supplemental Digital Content 1, http://links.lww.com/JS9/A103 and File 2, Supplemental Digital Content 2, http://links.lww.com/JS9/A104. The AMSTAR-2 checklist is available at Supplementary File 3, Supplemental Digital Content 3, http://links.lww.com/JS9/A105. This systematic review was prospectively registered in PROSPERO (Registration number CRD42022337384).

### Literature search

A literature search was conducted by one reviewer, followed by another reviewer. The MEDLINE, Embase and the Cochrane Library databases and the Cochrane Controlled Trials Register for RCTs were searched until February 2022. English literature was identified. The search term for MEDLINE is accessible in PROSPERO. References from previously published systematic reviews and selected studies were also searched.

### Inclusion and exclusion criteria

Studies were included if they: (1) were conducted in adult patients (>18 years old) diagnosed with hip fracture who received surgical treatment; (2) were RCTs; (3) compared the clinical outcome between general and neuraxial anaesthesia; and (4) reported at least one quantitative outcome in the research protocol. Studies were excluded if they: (1) used regional anaesthesia techniques other than neuraxial anaesthesia; (2) did not report outcomes in the research protocol; (3) did not report the number of patients on each arm; and (4) were conference abstracts, animal studies, cadaveric studies, in-vitro studies, or articles published in a form other than clinical trials.

### Study selection

Search results from the three databases were imported into the Endnote software. Duplicates were then removed. Two researchers, who were blinded to each other’s choices, screened studies according to the inclusion and exclusion criteria. First, each researcher screened the titles and abstracts of all references. The researcher then reviewed the full text of the articles selected in the previous step. The screening results from the two researchers were compared, and any disagreement was resolved by a third reviewer.

### Data extraction

The data was extracted using Microsoft Excel software (Microsoft, Redmond, WA, USA). One reviewer extracted the data and another reviewer examined it. Information extraction included study design, publication time, first author, methodology, patients’ demographic information, intervention details and clinical outcome. For discontinuous variables, events and rates were extracted. For continuous variables, the mean and SD were extracted. Unreported data or details were not extracted.

Outcomes reported in at least four RCTs were included. These included 30-day mortality, acute heart failure, myocardial infarction, delirium, cerebral vascular events, pneumonia, pulmonary embolism and postoperative nausea and vomiting (PONV). Subgroup analysis was performed based on patient age and the used of sedation in neuraxial anaesthesia. The quality of each included outcome was assessed by grading of recommendations, assessment, development and evaluations (GRADE). It assesses each outcome across different studies in terms of consistency, directness, precision, study design and risk of bias^27^,^28^.

### Risk of bias assessment

The risk of bias was independently appraised by two reviewers, and any disagreement was resolved by discussion with a third reviewer. RCTs were assessed using the Cochrane Collaboration’s tool for assessing the risk of bias in randomised trials^29^. It evaluates selection bias (random sequence generation and allocation concealment), performance bias (blinding of participants and personnel), detection bias (blinding of outcome assessment), attrition bias (incomplete outcome data), reporting bias (selective reporting) and other biases. Publication bias was assessed by using funnel plots. Meta-regression analysis was performed to detect the influence of publication year on the outcomes.

### Data synthesis

Review Manager software Version 5.3 (RevMan; Cochrane Collaboration, Oxford, UK) and R software Version 4.04 (R Core Team, Vienna, Austria) were used. The metafor package in R was used to conduct meta-regression analysis^30^. Discontinuous variables were analysed using odds ratios. The statistical methods and effect measures for each outcome are listed in Supplementary File 4, Supplemental Digital Content 4, http://links.lww.com/JS9/A106. A 95% CI was recorded for each outcome. Continuous variables were recorded as means and SDs. For studies which reported the median and interquartile range, the data were converted to the mean and SD according to the Cochrane Handbook for Systematic Reviews of Interventions^31^. The median and range were converted to mean and SD using the method described by Luo *et al*.^32^ and Wan *et al*.^33^ If SD was not reported, the average SD was used for imputation according to the Cochrane Handbook for Systematic Reviews of Interventions^31^. Heterogeneity among studies was assessed using *I* ^2^. When *I* ^2^ was greater than 0.5, a random effect model was applied. A fixed-effects model was applied when *I* ^2^ is less than 0.5. Statistical significance was set at *P* value less than 0.05. Due to the variation in sample size and publication time in the included studies, high between-study heterogeneity might be anticipated. An influence analysis was conducted by removing each study to further examine the results of the pooled analysis.

## Results

### Characteristics of included studies

**Figure 1:**
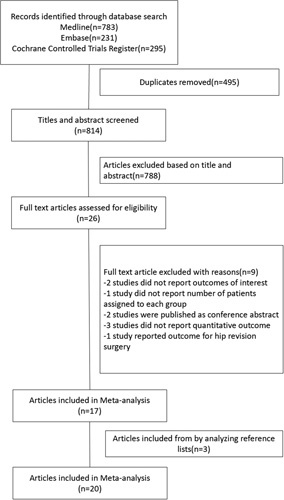
Flow diagram.

A total of 1309 studies were identified through a database search. A flow diagram of the screening process is shown in Figure 1. The full text of 26 articles was assessed for eligibility. Seventeen studies were included after full-text assessment, and three studies were included after the analysis of reference lists. Finally, 20 RCTs involving 4802 patients were included in this meta-analysis. The characteristics of the included studies are listed in Table 1. One study adopted spinal anaesthesia and a femoral nerve block in the neuraxial group^34^. One study adopted general anaesthesia and the lumbar-sacral plexus in the general anaesthesia group^17^. The details of the anaesthetic techniques used in each study are listed in Table 2.

**Table 1:** Characteristics of included studies.

		Participants			Outcome		
References	Country	Sample size	Age (years old)	ASA	Primary	Secondary	Follow up
Biboulet *et al.* 34	France	Total: 43 Neuraxial: 15 General: 28	>75 Neuraxial: 87 General: 85/86	III/IV	Hemodynamic data	Complications mortality	30 days
Bigler *et al*.^35^	Denmark	Total: 40 Neuraxial: 20 General: 20	>60	I/II/III	Mental function	Mortality Complication	90 days
Casati *et al*.^36^	Italy	Total: 30 Neuraxial: 15 General: 15	>65 Neuraxial: 84 General: 84	II/III	Hemodynamic data	Mental Function Complication	7 days
Davis *et al*.^37^	New Zealand	Total: 132 Neuraxial: 64 General: 68	>50 Neuraxial: 81 General: 78	III/IV: Neuraxial: 39 General: 44	Complication Mortality	Haemodynamic data	28 days
Davis *et al*.^38^	New Zealand	Total: 538 Neuraxial: 259 General: 279	>55	I/II/III/IV	Mortality	Complication	1 years
Haghighi *et al*.^39^	Iran	Total: 100 Neuraxial: 50 General: 50	>60 Neuraxial: 66 General: 66	I/II/III	Hemodynamic data	Blood loos Hospitalisation days Pain	PACU
Li *et al*.^40^	China	Total: 942 Neuraxial: 471 General: 471	>65 Neuraxial: 77 General: 77 (median)	I/II/III/IV	Delirium	Pain, Hospitalisation days, Mortality, Complication	30 days
Mckenzie *et al*.^41^	UK	Total: 100 Neuraxial: 51 General: 49	Neuraxial: 74.5 General: 76.8		Arterial oxygenation	Mortality	28 days
Mckenzie *et al*.^42^	UK	Total: 148 Neuraxial: 73 General: 75	Neuraxial: 75.4 General: 74.2		Mortality		1 year
Mckenzie *et al*.^43^	UK	Total: 40 Neuraxial: 20 General: 20	Neuraxial: 74 General: 72		Complication	Mortality	1 year
Meuret *et al.* 19	France	Total: 40 Neuraxial: 19 General: 21	>75 Neuraxial: 83 General: 85	I/II/III	Hemodynamic data	Complications Mortality Pain Surgeon satisfaction	30 days
Messina *et al*.^44^	Italy	Total: 20 Neuraxial: 10 General: 10	>75 Neuraxial: 82 General: 84	III	Hemodynamic data	Complications	
Neuman *et al*.^45^	USA	Total: 12 Neuraxial: 6 General: 6	>57		Delirium	Complications	
Neuman *et al.* 9	USA	Total: 1600 Neuraxial: 795 General: 805	>50 Neuraxial: 78 General: 78	I/II/III/IV	Mortality Morbidity	Complication Hospitalisation days	60 days
Parker and Griffiths 46	UK	Total: 332 Neuraxial: 158 General: 164	>49 Neuraxial: 82 General: 83	I/II: Neuraxial: 94 General: 98	Mortality	Complications	1 year
Shin *et al.* 18	Korea	Total: 176 Neuraxial: 58 General: 118	>65 Neuraxial: 82 General: 79/81		HMGB1 and IL-6	Morbidity Mortality	90 days
Simonin *et al.* 20	France	Total: 146 Neuraxial: 82 General: 64	>70 Neuraxial: 87.3 General: 86.5	I/II/III	Hemodynamic data	Complication	30 days
Tang *et al*.^47^	China	Total: 124 Neuraxial: 62 General: 62	>65 Neuraxial: 78 General: 77	I/II/III/IV	Morbidity	Pain Cost Complication	30 days
Tzimas *et al.* 16	Greece	Total: 70 Neuraxial: 77 General: 75	>65 Neuraxial: 77 General: 75	I/II/III	Cognitive Dysfunction	delirium	30 days
Wickstrom *et al*.^48^	Sweden	Total: 169 Neuraxial: 32 General: 137	>70 Epidural 83 General 78-82		Mortality		1 year

ASA, American Society of Anesthesiologists physical status classification system; general, general anaesthesia; HMGB1, high-mobility group box protein 1; IL-6, interleukin-6; neuraxial, neuraxial anaesthesia.

**Table 2:** Anaesthesia technique.

	Neuraxial anaesthesia				General anaesthesia		
References	Method	Medication	Sedation	Sedation medication	Induction	Airway	Maintenance
Biboulet *et al.* 34	Spinal	Bupivacaine 2.5 mg	None	None	Propofol/Sevoflurane	Intubation	Remifentanil
Bigler *et al*.^35^	Spinal	Bupivacaine 0.75% 3 ml	As needed	Unspecified	Fentanyl	Intubation	Fentanyl/N_2_O
Casati *et al*.^36^	Spinal	Bupivacaine 0.5% 7.5 mg	Unspecified	Unspecified	Sevoflurane	Mask	Sevoflurane
Davis *et al*.^37^	Spinal	Tetracaine/Cinchocaine	All	Diazepam	Diazepam/Fentanyl/N_2_O	Intubation	Fentanyl/N_2_O
Davis *et al*.^38^	Spinal	Tetracaine/Nupercaine/Bupivacaine	As needed	Benzodiazepine	Thiopentone	Intubation	Fentanyl/N_2_O
Haghighi *et al*.^39^	Spinal	Lidocaine 75 mg	Unspecified	Unspecified	Fentanyl/Propofol	Intubation	Sevoflurane/N_2_O
Li *et al*.^40^	Spinal/Epidural	Chosen by anaesthesiologist	None	None	Unspecified	Intubation/Mask	Unspecified
Mckenzie *et al*.^41^	Spinal	0.5% Cinchocaine 1.3–1.5 ml	All	Diazepam	Althesin	Intubation	N_2_O/Halothane
Mckenzie *et al*.^42^	Spinal	0.5% Cinchocaine 1.3–1.5 ml	As needed	Diazepam	Althesin	Intubation	N_2_O/Halothane
Mckenzie *et al*.^43^	Spinal	Cinchocaine 1.2–1.5 ml	As needed	Diazepam	Althesin	Intubation	N_2_O/Halothane
Meuret *et al.* 19	Spinal	Bupivacaine 5 mg/Sufentanil 5 µg	None	None	Remifentanil	Intubation	Remifentanil
Messina *et al*.^44^	Spinal	Levobupivacaine 7.5 mg/Sufentanil 5 µg	All	Unspecified	Remifentanil/Propofol	Intubation	Sevoflurane
Neuman *et al*.^45^	Spinal	Chosen by anaesthesiologist					
Neuman *et al.* 9	Spinal	Unspecified	As needed	Unspecified	Chosen by anaesthesiolgist	Inhaled agent	
Parker and Griffiths 46	Spinal	Chosen by anaesthesiologist					
Shin *et al.* 18	Spinal	Bupivacaine 9 mg/1 mg	As needed	Unspecified	Remifentanil/Pentothal sodium/Propofol	Intubation	Desflurane/Remifentanil/Propofol
Simonin *et al.* 20	Spinal	Ropivacaine 9 mg and Sufentanil 5 µg)	As needed	Remifentanil	Etomidate and remifentanil	Intubation	Desflurane and Remifentanil
Tang *et al*.^47^	Spinal	Ropivacaine 4 ml 0.25%	All	Propofol	Propofol/Sufentanil	Mask	Propofol
Tzimas *et al.* 16	Spinal	Fentanyl 20mcg/Ropivacaine 0.75%	None	None	Fentanyl: 3–5 µg/kg/Propofol: 1.5 mg/kg	Intubation	Desflurane
Wickstrom *et al*.^48^	Epidural	Mepivacaine	All	Droperidol	Ketamine/Neurolept/Enflurane/Halothane	Intubation	Ketamine/Neurolept/Enflurane/Halothane

Epidural, epidural anaesthesia; N_2_O, nitrous oxide; spinal, spinal anaesthesia.

Eighteen of the 20 studies had an unclear risk of blinding of participants and personnel due to a lack of relevant information. Three studies had a high risk of incomplete data. The risks of bias in random sequence generation, allocation concealment and blinding of outcome assessment were high in two studies. One study had a high risk of bias in selective reporting. The results of the risk of bias assessment are presented in Supplementary File 5, Supplemental Digital Content 5, http://links.lww.com/JS9/A107 and Supplementary File 6, Supplemental Digital Content 6, http://links.lww.com/JS9/A108.

### Data synthesis

The quality of each outcome variable, as assessed by GRADE, is listed in Supplementary File 7, Supplemental Digital Content 7, http://links.lww.com/JS9/A109. The outcomes included postoperative mortality, delirium, PONV, myocardial infarction, acute heart failure, pneumonia, pulmonary embolism, cerebrovascular accidents and AKI. Meta-regression was performed for each outcome variable to determine whether the year of publication had a significant influence on the risk of adverse events. Subgroup analysis was performed based on patient age and the use of sedation during neuraxial anaesthesia. Subgroup analysis based on patient age was performed at 5-year intervals. The length of hospital stay and duration of surgery were also investigated.

**Figure 2:**
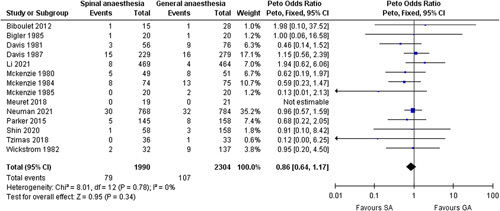
Forest plot of postoperative mortality. GA, general anaesthesia; SA, spinal anaesthesia.

**Figure 3:**
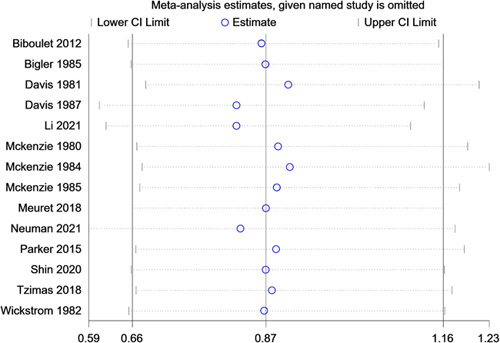
Influence analysis of postoperative mortality.

Data synthesis revealed a significantly higher risk of postoperative AKI in patients receiving general anaesthesia group (*P*=0.01). There were no significant differences in postoperative mortality (*P*=0.29), delirium (*P*=0.40), pneumonia (*P*=0.15), PONV (*P*=0.63), myocardial infraction (*P*=0.22), cerebral vascular accident (*P*=0.60) and heart failure (*P*=0.34) between the two anaesthetic techniques. The forest plot of mortality was shown in Figure 2. The results were shown in Table 3. Sensitivity analysis was performed, which only included data from literature published within the time frame of 20 years. Similar results were obtained and are shown in Table 4. Influence analysis of mortality did not reveal significant heterogeneity in any of the included studies (Fig. 3).

**Table 3:** Results from data synthesis.

Outcome	Number of studies included	OR	95% CI	*P*	*I* ^2^
Death	15	0.95	0.20–4.50	0.29	0
Delirium	7	1.10	0.88–1.38	0.40	0
PONV	6	0.73	0.20–2.67	0.63	0.55
Myocardial infarction	6	0.6	0.27–1.35	0.22	0
Pneumonia	6	0.65	0.36–1.17	0.15	0
Cerebral vascular accident	7	0.8	0.36–1.81	0.60	0.36
Pulmonary embolism	5	0.57	0.22–1.47	0.24	0
Heart failure	4	0.58	0.19–1.76	0.34	0
Acute kidney injury	4	0.58	0.39–0.88	0.01	0
Outcome	Number of studies included	SMD	95% CI	*P*	*I* ^2^
Hospital stay	8	0.04	−0.04 to 0.12	0.37	0.46
Duration of surgery	11	0.65	0.32–0.97	0.70	0.96

OR, odds ratio; PONV, postoperative nausea and vomiting; SMD, standard mean difference.

**Table 4:** Data synthesis based on studies published within 20 years.

Outcome	Number of studies included	OR	95% CI	*P*	*I* ^2^
Death	8	0.96	0.64–1.43	0.83	0
Delirium	7	1.1	0.88–1.38	0.4	0
PONV	5	0.66	0.23–1.9	0.44	0.66
Myocardial infarction	5	0.63	0.25–1.55	0.31	0
Pneumonia	3	0.52	0.24–1.09	0.08	0
Cerebral vascular accident	4	0.64	0.21–1.89	0.42	0
Pulmonary embolism	2	0.59	0.18–1.92	0.38	0.21
Heart failure	3	0.62	0.13–3.02	0.55	0.7
Acute kidney injury	4	0.58	0.39–0.88	0.01	0
Outcome	Number of studies included	SMD	95% CI	*P*	*I* ^2^
Hospital stay	6	0.04	−0.06 to 0.13	0.43	0.56
Duration of surgery	7	−0.53	−1.33 to 0.27	0.19	0.94

OR, odds ratio; PONV, postoperative nausea and vomiting; SMD, standard mean difference.

Subgroup analysis was performed based on the inclusion criteria of patient age in each study (>75, >70, >65, >60, >55 and >50 years) (Supplementary File 8, Supplemental Digital Content 8, http://links.lww.com/JS9/A110) and the use of sedation in neuraxial anaesthesia (Supplementary File 9, Supplemental Digital Content 9, http://links.lww.com/JS9/A111). The results did not reveal any significant difference in the risk of adverse events, including mortality, delirium, PONV, pneumonia and cerebrovascular accidents.

**Figure 4:**
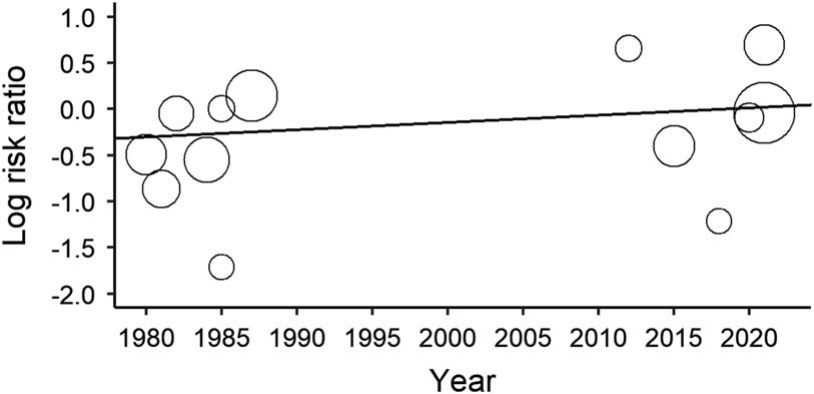
Meta-regression analysis of postoperative mortality versus publication year.

Meta-regression of the publication year versus each adverse event revealed no statistically significant differences in mortality (*P*=0.364), delirium (*P*=0.249), PONV (*P*=0.979), myocardial infarction (*P*=0.914), acute heart failure (*P*=0.731), pneumonia (*P*=0.304), pulmonary embolism (*P*=0.872), cerebral vascular accident (*P*=0.859), or AKI (*P*=0.748). The results were shown in Figure 4. This suggested that the risk of adverse events did not change significantly with the publication year.

## Discussion

The results of this study showed a significantly higher risk of AKI in patients receiving general anaesthesia. There was no significant difference between neuraxial and general anaesthesia in terms of postoperative mortality, length of hospital stay, duration of surgery and other complications. According to the GRADE assessment, moderate to high certainty was achieved in outcomes including mortality, delirium, pneumonia and AKI.

It has been argued that general anaesthesia may be associated with an increased risk of postoperative complications due to intubation and the use of anaesthetics^49^. Intubation may be a risk factor for developing pneumonia^50^. The use of intravenous and inhaled anaesthetics may influence the risk of delirium^51^. However, the results of this study, as well as some other studies, have revealed no significant differences in postoperative complications^9^,^14^,^52^. Anaethesia type is one of the many factors that may influence the risk of postoperative complications. The similar postoperative complication rate might be explained by the fact that the patients received the same standardized perioperative management in the RCTs. Concurrent perioperative management allowed for early mobilisation, optimal pain management and early functional recovery, which may significantly reduce the risk of postoperative complications^53^,^54^. Second, prolonged surgical time is another important risk factor for postoperative complications^55^,^56^. However, the results of data synthesis suggested that anaesthesia type did not significantly influenced the duration of surgery and the mean surgery time was under 90 min for most studies. Lastly, it has been reported that sevoflurane may induce or exacerbate neuroinflammation, which could potentially increase the risk of delirium^57^. In this systematic review, we summarised the anaesthetics used in each study, 16 out of the 20 studies did not use sevoflurane. Therefore, different anaesthetics and techniques used in general anaesthesia might be another reason for this finding.

Pu and Sun^58^ performed a systematic review and meta-analysis to investigate the optimal anaesthetic technique for patients receiving total hip arthroplasty. They found that spinal anaesthesia was associated with a lower risk of nausea and a shorter hospital stay. The differences in findings might be explained by the difference in patient population between their study and ours. Other than trauma, total hip arthroplasty is also indicated in developmental dysplasia of hip, avascular necrosis of the femoral head, rheumatoid arthritis, ankylosing spondylitis and other medical conditions affecting the hip joint^59^,^60^. On the other hand, total hip arthroplasty is only one of the surgical treatments for hip fracture; other surgical procedures included femoral head replacement and internal fixation^61^. Therefore, the patients’ demographic characteristics, primary diagnosis, reasons for surgery and surgical procedures might be different between the two studies, which led to different results. Additionally, revision arthroplasty was included in the study conducted by Pu and Sun^58^. Revision hip arthroplasty is a more complex surgical procedure than primary arthroplasty with significantly prolonged surgical times and a higher risk of complications^62^. Previous reviews focusing on patients receiving hip fracture surgery reached conclusions similar to ours^2^,^8^.

In previous reviews, articles published prior to 2000 were excluded to focus on the influence of modern anaesthetic technique^2^,^8^. However, in studies published in different time periods, the differences in anaesthetic techniques remain unknown. The reason for choosing the year 2000 as the criterion for inclusion also remains unclear. Therefore, we included studies from all available time periods and compared the anaesthetic techniques (Table 2). Meta-regression was performed to investigate the influence of the publication year on each outcome. The results showed that publication year did not have a significant correlation with the risks of mortality and other complications, suggesting that evidence from studies published prior to 2000 did not introduce bias to data synthesis.

Older patients are at higher risk of postoperative mortality and other complications after hip fracture surgery^63^,^64^. A previous systematic review investigated the outcome in patients aged greater than or equal to 60 years and found no evidence suggesting that the type of anaesthesia influenced postoperative delirium^21^. Subgroup analysis was performed to compare the risk of each adverse event in senior patients. According to the inclusion criteria of each trial, six subgroups were established: age above 50 years, above 55 years, above 60 years, above 65 years, above 70 years and above 75 years. The risks of postoperative mortality and other complications were similar between neuraxial and general anaesthesia in all subgroup analyses based on patient age.

The influence of sedative medication on postoperative delirium and PONV has been reported in previous studies^65^,^66^. A subgroup analysis was conducted based on the use of sedation during neuraxial anaesthesia. The results showed no significant difference between the two anaesthetic techniques, regardless of the use of sedation.

To a certain extent, the duration of surgery reflects its technical difficulty. Prolonged surgery duration increases the risk of major and minor postoperative complications^67^,^68^. In neuraxial anaesthesia without sedation, the patients were awake and subjected to psychological stress and abnormal sensations, which may affect the progress of surgery and postoperative recovery. However, there was no significant difference in the length of stay and duration of surgery between neuraxial and general anaesthesia.

Most trials in this field have focused on postoperative mortality and other complications. Few studies have investigated the influence of anaesthetic techniques on functional recovery following hip fracture surgery^17^. Tang *et al*.^17^ reported that the daily living scale was comparable between the anaesthetic techniques 30 days after surgery. Neuman *et al*.^9^ reported whether patients were able to walk 60 days after surgery. Early mobilisation within 48 hours after surgery has become a common practise in these patients, which is more favourable than delayed mobilisation in terms of complications and functional outcome^69^,^70^. Further studies are required to investigate whether the two anaesthetic techniques influence the outcomes of early mobilisation.

The strengths of this study are as follows: only randomized controlled trials were included in this study, which avoided selection bias. Comprehensive subgroup analysis was performed, which is based on patient age (>75, >70, >65, >60, >55 and >50 years) and anaesthetic technique. GRADE assessment showed moderate to high certainty for primary outcomes. Meta-regression analysis was performed to evaluate the influence of publication time on each outcome.

The limitations of this study are as follows: other than mortality, the outcome definition varied among included studies, which may cause bias in data synthesis. Details of methodology are not fully specified in a proportion of studies, which are rated as having an unclear risk by the Cochrane Collaboration’s tool for assessing the risk of bias. Hip fracture surgeries included internal fixation and arthroplasty. The type of surgery may influence the clinical outcome as well. However, the type of surgery was expected to be evenly distributed between groups through randomisation. This is also why only randomised controlled trials were included, to avoid physicians’ preferences to adopt certain types of anaesthesia for certain type of procedures.

## Conclusion

A significantly higher risk of postoperative AKI was found in patients receiving general anaesthesia. This study revealed no significant differences in terms of postoperative mortality and other complications between general and neuraxial anaesthesia. The results were consistent across the age groups.

## Article focus

1. Determine the optimal anaesthetic technique for hip fracture surgery through a systematic review.

2. Compare the risk of complication and mortality between general anaesthesia and neuraxial anaesthesia in patients across all age groups.

3. Investigate the influence of anaesthetic technique on the risk of complications and mortality.

## Key messages

1. Nineteen randomised controlled trials were included in the data synthesis.

2. A significantly higher risk of postoperative acute kidney injury was found in patients receiving general anaesthesia.

3. There were no significant differences in terms of postoperative mortality and other complications between general and neuraxial anaesthesia.

## Strengths and limitations

1. Only randomised controlled trials were included in this study, which avoided selection bias.

2. A comprehensive subgroup analysis based on patient age (>75, >70, >65, >60, >55 and >50 years) and anaesthetic technique was performed.

3. Grading of recommendations, assessment, development and evaluations assessment showed moderate to high certainty for primary outcomes. Meta-regression analysis was performed to evaluate the influence of publication time on each outcome.

4. There were variations in outcome definition and reporting timing across included studies.

5. Details of methodology are not fully specified in some studies, which are rated as having an unclear risk by the Cochrane Collaboration's tool for assessing the risk of bias.

## Ethical approval

Since our study was a meta-analysis, an ethical review committee statement was not required.

## Sources of funding

Key Research and Development Fund of Sichuan Science and Technology Planning Department (grant number2022YFS0372). Youth Research Fund of Sichuan Science and Technology Planning Department (grant number 23NSFSC4894).

## Author contribution

X.C., W.W.Q. and J.L. came up with this research topic and analysed the data. X.C. wrote the manuscript. H.L., S.L. and Y.W. searched the databases and retrieved data from the included studies. R.M. and G.C. searched the databases, checked the included articles, retrieved the data and recorded them. X.C. and G.C. developed and checked methods for rigorousity. H.L. and X.C. edited the language and performed statistical analysis of this article. All authors have read and approved the final version of the manuscript.

## Conflicts of interest disclosure

None.

## Research registration unique identifying number (UIN)

1. Name of the registry: PROSPERO.
2. Unique identifying number or registration ID: CRD42022337384.
3. Hyperlink to your specific registration (must be publicly accessible and will be checked): https://www.crd.york.ac.uk/PROSPERO/display_record.php?RecordID=337384

## Guarantor

Xi Chen.

## Data availability statement

Since our study was a meta-analysis, all data are available and published by the studies included.

## Supplementary Material
