# Supplementary material for: Comparison of risk of complication between neuraxial anaesthesia and general anaesthesia for hip fracture surgery: a systematic review and meta-analysis
Source: Int J Surg. 2023 Mar 24;109(3):458–68. doi: 10.1097/JS9.0000000000000291 (PMC10389547; doi:10.1097/JS9.0000000000000291)
Supplement: Supplementary file 2 [file js9-109-458-s002.docx]

**Identification of new studies via other methods**

**Previous studies**

**Identification of new studies via databases and registers**

Studies included in previous version of review (n = 9)

Reports of studies included in previous version of review (n = 9)

Records identified from*:

Databases (n =1309 )

Medline(n=783)

Embase(n=231)

Cochrane Controlled Trials Register(n=295)

Records removed *before screening*:

Duplicate records removed (n = 495)

Records marked as ineligible by automation tools (n = 0)

Records removed for other reasons (n = 0)

Records identified from:

Websites (n = 0)

Organisations (n = 0)

Citation searching (n =3 )

etc.

**Identification**

Total studies included in review

(n = 20)

Reports assessed for eligibility

(n = 26)

Reports sought for retrieval

(n = 26)

Records screened

(n = 814)

Records excluded**

(n = 788)

Reports not retrieved

(n = 0)

Reports sought for retrieval

(n = 3)

Reports not retrieved

(n = 0)

**Screening**

Reports excluded (n=9)

-2 studies did not report outcomes of interest

-1 study did not report number of patients assigned to each group

-2 studies were published as conference abstract

-3 studies did not report quantitative outcome

-1 study reported outcome for hip revision surgery

Reports excluded: 0

Reports assessed for eligibility

(n = 3)

New studies included in review

(n = 11)

**Included**

*Consider, if feasible to do so, reporting the number of records identified from each database or register searched (rather than the total number across all databases/registers).

**If automation tools were used, indicate how many records were excluded by a human and how many were excluded by automation tools.

From: Page MJ, McKenzie JE, Bossuyt PM, Boutron I, Hoffmann TC, Mulrow CD, et al. The PRISMA 2020 statement: an updated guideline for reporting systematic reviews. BMJ 2021;372:n71. doi: 10.1136/bmj.n71. For more information, visit: <http://www.prisma-statement.org/>
