# Supplementary material for: Comparison of risk of complication between neuraxial anaesthesia and general anaesthesia for hip fracture surgery: a systematic review and meta-analysis
Source: Int J Surg. 2023 Mar 24;109(3):458–68. doi: 10.1097/JS9.0000000000000291 (PMC10389547; doi:10.1097/JS9.0000000000000291)
Supplement: Supplementary file 4 [file js9-109-458-s004.docx]

Supplementary Table 1. Statistical methods adopted in each outcome analysis

| Outcome Variable | Statistical Method | Effect Measure | Analysis Model |
| --- | --- | --- | --- |
| Mortality | Peto | Peto OR | Fixed Effect |
| Acute Heart Failure | Peto | Peto OR | Fixed Effect |
| Myocardial Infarction | Peto | Peto OR | Fixed Effect |
| Pneumonia | Mantel-Haenszel | OR | Fixed Effect |
| Pulmonary Embolism | Peto | Peto OR | Fixed Effect |
| Cerebral Vascular Accident | Peto | Peto OR | Fixed Effect |
| Acute kidney injury | Peto | Peto OR | Fixed Effect |
| PONV | Mantel-Haenszel | OR | Random Effect |
| Delirium | Mantel-Haenszel | OR | Fixed Effect |
| Length of Hospital Stay | Inverse Variance | Std. Mean Difference | Fixed Effect |
| Duration of Surgery | Inverse Variance | Std. Mean Difference | Random Effect |
