# Supplementary figures and images for: Comparison of risk of complication between neuraxial anaesthesia and general anaesthesia for hip fracture surgery: a systematic review and meta-analysis
Source: Int J Surg. 2023 Mar 24;109(3):458–68. doi: 10.1097/JS9.0000000000000291 (PMC10389547; doi:10.1097/JS9.0000000000000291)

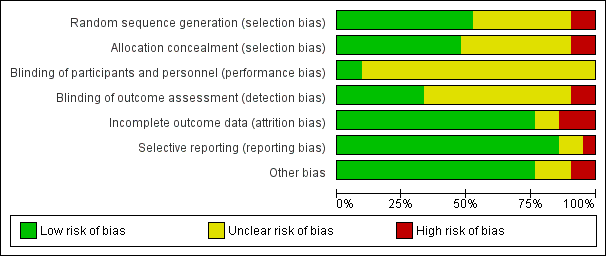

Supplement: Supplementary file 5 [file js9-109-458-s005.jpg]

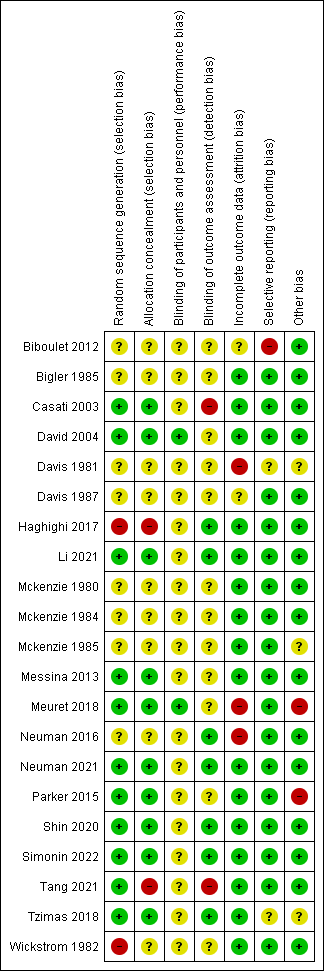

Supplement: Supplementary file 6 [file js9-109-458-s006.jpg]
