# Supplementary material for: Comparison of risk of complication between neuraxial anaesthesia and general anaesthesia for hip fracture surgery: a systematic review and meta-analysis
Source: Int J Surg. 2023 Mar 24;109(3):458–68. doi: 10.1097/JS9.0000000000000291 (PMC10389547; doi:10.1097/JS9.0000000000000291)
Supplement: Supplementary file 7 [file js9-109-458-s007.docx]

Supplementary File 7. Grade Assessment of Each Outcome

**Author(s):**

**Question:** SA compared to GA for surgery for hip fractures in elderly

**Setting:**

**Bibliography:** , , , , . GA versus SA for surgery for hip fractures in elderly. Cochrane Database of Systematic Reviews [Year], Issue [Issue].

| **Certainty assessment** | | | | | | | **№ of patients** | | **Effect** | | **Certainty** | **Importance** |
| --- | --- | --- | --- | --- | --- | --- | --- | --- | --- | --- | --- | --- |
| **№ of studies** | **Study design** | **Risk of bias** | **Inconsistency** | **Indirectness** | **Imprecision** | **Other considerations** | **SA** | **GA** | **Relative (95% CI)** | **Absolute (95% CI)** |  |  |
| **Mortality** | | | | | | | | | | | | |
| 15 | randomised trials | serious | not serious | not serious | not serious | none | 81/2072 (3.9%) | 110/2368 (4.6%) | **OR 0.85** (0.63 to 1.15) | **7 fewer per 1,000** (from 17 fewer to 7 more) | ⨁⨁⨁◯ Moderate | CRITICAL |
| **Delirium** | | | | | | | | | | | | |
| 7 | randomised trials | not serious | not serious | not serious | not serious | all plausible residual confounding would reduce the demonstrated effect | 186/1403 (13.3%) | 179/1470 (12.2%) | **OR 1.10** (0.88 to 1.38) | **11 more per 1,000** (from 13 fewer to 39 more) | ⨁⨁⨁⨁ High | CRITICAL |
| **Postoeprative nausea and vomit(PONV)** | | | | | | | | | | | | |
| 6 | randomised trials | serious | serious^a^ | not serious | serious^b^ | none | 60/626 (9.6%) | 61/572 (10.7%) | **OR 0.73** (0.20 to 2.67) | **26 fewer per 1,000** (from 83 fewer to 135 more) | ⨁◯◯◯ Very low | IMPORTANT |
| **Myocardial infarction** | | | | | | | | | | | | |
| 6 | randomised trials | serious | not serious | not serious | serious^c^ | none | 9/1512 (0.6%) | 15/1526 (1.0%) | **OR 0.60** (0.27 to 1.35) | **4 fewer per 1,000** (from 7 fewer to 3 more) | ⨁⨁◯◯ Low | IMPORTANT |
| **Pneumonia** | | | | | | | | | | | | |
| 6 | randomised trials | serious | not serious | not serious | not serious | none | 18/1562 (1.2%) | 29/1599 (1.8%) | **OR 0.65** (0.36 to 1.17) | **6 fewer per 1,000** (from 12 fewer to 3 more) | ⨁⨁⨁◯ Moderate | IMPORTANT |
| **Cerebral vascular accident** | | | | | | | | | | | | |
| 7 | randomised trials | serious | not serious | not serious | serious^c^ | none | 10/1773 (0.6%) | 14/1880 (0.7%) | **OR 0.80** (0.36 to 1.81) | **1 fewer per 1,000** (from 5 fewer to 6 more) | ⨁⨁◯◯ Low | IMPORTANT |
| **Pulmonary embolism** | | | | | | | | | | | | |
| 5 | randomised trials | serious | not serious | not serious | serious^c^ | none | 6/1264 (0.5%) | 11/1331 (0.8%) | **OR 0.57** (0.22 to 1.47) | **4 fewer per 1,000** (from 6 fewer to 4 more) | ⨁⨁◯◯ Low | IMPORTANT |
| **Heart failure** | | | | | | | | | | | | |
| 4 | randomised trials | serious | serious^d^ | not serious | very serious^c^ | none | 4/700 (0.6%) | 10/739 (1.4%) | **OR 0.58** (0.19 to 1.76) | **6 fewer per 1,000** (from 11 fewer to 10 more) | ⨁◯◯◯ Very low | IMPORTANT |
| **Duration of surgery** | | | | | | | | | | | | |
| 11 | randomised trials | not serious | serious^e^ | not serious | not serious | publication bias strongly suspected^a^ | 412 | 425 | - | SMD **0.14 SD lower** (0.86 lower to 0.58 higher) | ⨁⨁◯◯ Low |  |
| **Length of hospital stay** | | | | | | | | | | | | |
| 7 | randomised trials | not serious | serious^a^ | not serious | not serious | publication bias strongly suspected^f^ | 1098 | 1191 | - | SMD **0.01 SD higher** (0.07 lower to 0.1 higher) | ⨁⨁◯◯ Low |  |
| **Acute kidney injury (follow-up: 10 years)** | | | | | | | | | | | | |
| 1 | randomised trials | not serious | not serious | not serious | serious^g^ | none | 36/1005 (3.6%) | 61/1030 (5.9%) | **OR 0.59** (0.44 to 0.79) | **23 fewer per 1,000** (from 32 fewer to 12 fewer) | ⨁⨁⨁◯ Moderate |  |

**CI:** confidence interval; **OR:** odds ratio; **SMD:** standardised mean difference

#### Explanations

a. Inconsistent results were reported

b. Wide confidence interval showed in pooled analysis.

c. The number of events observed was small

d. Event rate was high variable across different studies

e. The definition of surgery time is inconsistent among studies

f. Funnel plot suggested publication bias

g. Diagnostic criteria was not fully explained by some studies
