# Supplementary material for: Comparison of risk of complication between neuraxial anaesthesia and general anaesthesia for hip fracture surgery: a systematic review and meta-analysis
Source: Int J Surg. 2023 Mar 24;109(3):458–68. doi: 10.1097/JS9.0000000000000291 (PMC10389547; doi:10.1097/JS9.0000000000000291)
Supplement: Supplementary file 8 [file js9-109-458-s008.docx]

Supplementary File 8. Subgroup Analysis of Adverse Event Based on Inclusion Age of Randomized Trials

| Outcome | Subgroup | No. of studies included | OR | 95%CI | P | I^2^ |
| --- | --- | --- | --- | --- | --- | --- |
| Mortality |  |  |  |  |  |  |
|  | >50 years | 10 | 1.04 | 0.73-1.49 | 0.84 | 0 |
|  | >55 years | 9 | 1.13 | 0.68-1.87 | 0.64 | 0 |
|  | >60 years | 8 | 1.11 | 0.55-2.24 | 0.78 | 0 |
|  | >65 years | 7 | 1.11 | 0.51-2.31 | 0.77 | 0 |
|  | >70 years | 4 | 0.83 | 0.28-2.48 | 0.74 | 0 |
| Delirium |  |  |  |  |  |  |
|  | >50 years | 6 | 1.08 | 0.86-1.36 | 0.51 | 0 |
|  | >55 years | 5 | 1.14 | 0.76-1.71 | 0.52 | 0 |
|  | >65 years | 4 | 1.18 | 0.79-1.78 | 0.42 | 0 |
| PONV |  |  |  |  |  |  |
|  | >65 years | 4 | 0.96 | 0.40-2.34 | 0.93 | 51% |
| Myocardial Infarction | | |  |  |  |  |
| Not Applicable | | | | | | |
| Heart Failure | |  |  |  |  |  |
| Not Applicable | | | | | | |
| Cerebral Vascular Accident | | |  |  |  |  |
|  | >50 years | 5 | 0.87 | 0.38-1.99 | 0.74 | 45% |
| Pneumonia | |  |  |  |  |  |
|  | >50 years | 4 | 0.51 | 0.25-1.04 | 0.07 | 0% |
| Pulmonary embolism | |  |  |  |  |  |
| Not Applicable | | | | | | |

Subgroup analysis was not performed if less than four studies were included
