# Supplementary material for: Comparison of risk of complication between neuraxial anaesthesia and general anaesthesia for hip fracture surgery: a systematic review and meta-analysis
Source: Int J Surg. 2023 Mar 24;109(3):458–68. doi: 10.1097/JS9.0000000000000291 (PMC10389547; doi:10.1097/JS9.0000000000000291)
Supplement: Supplementary file 9 [file js9-109-458-s009.docx]

Supplementary File 9.Subgroup Analysis of Whether Sedative Medication Was Used in Neuraxial Group

| Outcome | Subgroup | No. of studies included | OR | 95%CI | P | I^2^ |
| --- | --- | --- | --- | --- | --- | --- |
| Mortality |  |  |  |  |  |  |
|  | Used | 10 | 0.82 | 0.59-1.13 | 0.36 | 0 |
|  | Not used | 4 | 1.61 | 0.58-4.49 | 0.36 | 0 |
| Delirium |  |  |  |  |  |  |
|  | Not Applicable | |  |  |  |  |
| PONV |  |  |  |  |  |  |
|  | Not Applicable | |  |  |  |  |
| Heary Failure | |  |  |  |  |  |
|  | Not Applicable | |  |  |  |  |
| Myocardial Infarction | | |  |  |  |  |
|  | Not Applicable | |  |  |  |  |
| Cerebral Vascular Accident | | |  |  |  |  |
|  | Used | 4 | 0.87 | 0.38-1.99 | 0.74 | 45% |
| Pneumonia | |  |  |  |  |  |
|  | Not Applicable | |  |  |  |  |
| Pulmonary embolism | |  |  |  |  |  |
|  | Used | 4 | 0.68 | 0.25-1.89 | 0.46 | 0% |

Subgroup analysis was not performed if less than four studies were included
